# Delayed Hemorrhage Following Needle Aspiration for a Mediastinal Cyst: The Significance of Confronting Upside-Down Video-Assisted Thoracic Surgery Under Two-Lung Ventilation

**DOI:** 10.70352/scrj.cr.25-0134

**Published:** 2025-04-29

**Authors:** Eri Ueda, Tomonari Oki, Shuhei Iizuka, Yoshifumi Kunii, Yoshiro Otsuki, Toru Nakamura

**Affiliations:** 1 Department of General Thoracic Surgery, Seirei Hamamatsu General Hospital, Hamamatsu, Shizuoka, Japan; 2 Department of Cardiovascular Surgery, Seirei Hamamatsu General Hospital, Hamamatsu, Shizuoka, Japan; 3 Department of Pathology, Seirei Hamamatsu General Hospital, Hamamatsu, Shizuoka, Japan

**Keywords:** mediastinal neoplasms, thoracic surgery, video-assisted, extracorporeal membrane oxygenation

## Abstract

**INTRODUCTION:**

Surgical resection remains the gold standard for managing mediastinal cysts, including bronchogenic cysts, whereas needle aspiration serves as an alternative option that can facilitate preoperative volume reduction or, in certain selected cases, serve as a definitive treatment. However, it may lead to rare but potentially life-threatening complications such as mediastinitis; therefore, its indication should be carefully considered. This report details a case of a delayed intracystic hemorrhage 3 days after an endoscopic ultrasound-guided fine-needle aspiration (EUS-FNA), requiring emergency surgery with venoarterial extracorporeal membrane oxygenation (V-A ECMO) on standby, which was successfully managed using a confronting upside-down video-assisted thoracoscopic surgery (VATS) approach.

**CASE PRESENTATION:**

A 64-year-old woman with exertional dyspnea was diagnosed with a superior mediastinal cyst compressing the trachea and esophagus. Preoperative EUS-FNA was performed to reduce the cyst volume and any mitigate potential complications during anesthesia induction. Three days later, she developed dyspnea due to a delayed intracystic hemorrhage, necessitating emergency surgery. VATS with a confronting upside-down monitor setup was performed under standby V-A ECMO. Despite a limited surgical field under 2-lung ventilation, a confronting upside-down VATS approach allowed sufficient visualization and maneuverability. The patient had an uneventful recovery, with no recurrence at 3 months.

**CONCLUSIONS:**

A delayed intracystic hemorrhage is a potential risk following an EUS-FNA for mediastinal cysts. A confronting upside-down VATS approach provides sufficient maneuverability even for superior mediastinal tumors, despite a limited surgical field due to inadequate 1-lung ventilation. Placement of the camera port in the higher intercostal space was deemed particularly crucial.

## Abbreviations

ESGE
: European Society of Gastrointestinal Endoscopy
EUS-FNA
: endoscopic ultrasound-guided fine-needle aspiration
V-A ECMO
: venoarterial extracorporeal membrane oxygenation
VATS
: video-assisted thoracoscopic surgery

## INTRODUCTION

Most mediastinal cysts, including bronchogenic cysts, are often asymptomatic but can present with symptoms due to bleeding, infection, or compression of surrounding structures such as the esophagus, trachea, and bronchi.^1–3)^ While a complete surgical resection remains the gold standard treatment, alternative approaches such as a puncture aspiration have also been reported, which may serve as a definitive treatment in selected cases or necessitate subsequent surgery.^3–8)^ However, a puncture aspiration carries a risk of a cyst recurrence and may also lead to procedure-related complications.^9–11)^

We present a case of a mediastinal cyst requiring emergency surgery with V-A ECMO on standby due to a delayed intracystic hemorrhage after an EUS-FNA.

## CASE PRESENTATION

**Fig. 1:**
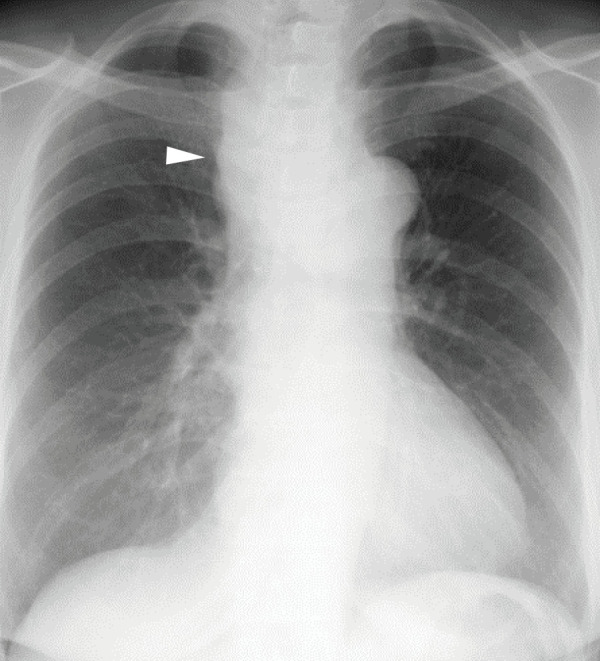
A chest radiograph showing a widened mediastinum (arrowhead).

**Fig. 2:**
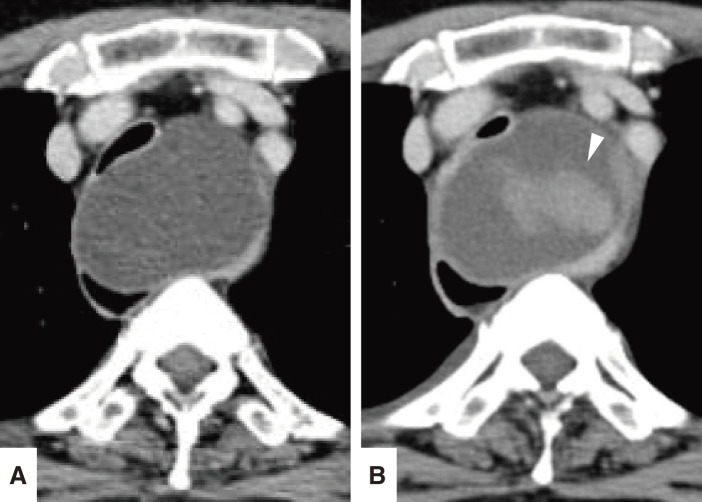
(**A**) Initial contrast-enhanced CT image showing a simple mediastinal cyst between the trachea and the esophagus. (**B**) Contrast-enhanced CT 3 days after the EUS-FNA showing contrast enhancement within the mass, suggesting an intracystic hemorrhage (arrowhead). EUS-FNA, endoscopic ultrasound-guided fine-needle aspiration

**Fig. 3:**
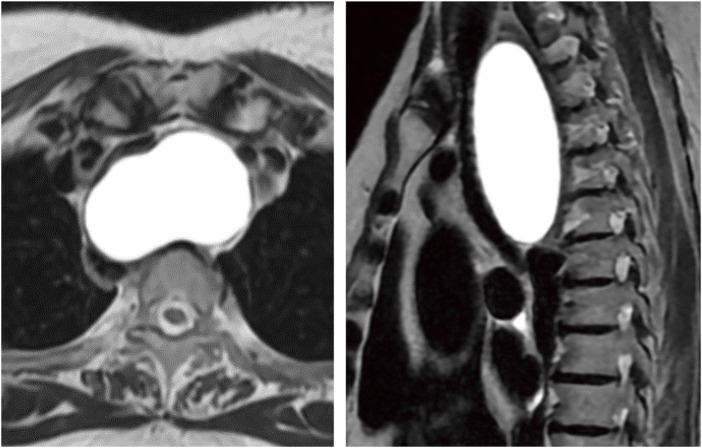
MRI showing a homogeneously hyperintense signal mass in the superior mediastinum on the T2-weighted images.

A 64-year-old woman presented with progressively worsening exertional dyspnea. She had no significant medical history and was not taking any oral medications, including anticoagulants. Laboratory evaluation revealed a hemoglobin level of 13.1 g/dL and a platelet count of 28.2 × 10^4^/mm^3^. The prothrombin time was 10.1 seconds, and the international normalized ratio was 0.87. The activated partial-thromboplastin time was 26.5 seconds (normal, <34). A chest radiograph showed a widened mediastinum (**Fig. 1**), and contrast-enhanced CT revealed a well-demarcated oval mass expanding between the membranous portion of the trachea and the esophagus (**Fig. 2A**). The lesion exhibited homogeneous low attenuation, measuring 16–20 Hounsfield units, with no evidence of contrast enhancement. The wall was thin, and no solid components or septations were observed, supporting the diagnosis of a simple mediastinal cyst. Contrast-enhanced MRI showed a uniformly high-signal intensity on T2-weighted images, which was consistent with the CT findings (**Fig. 3**).

**Fig. 4:**
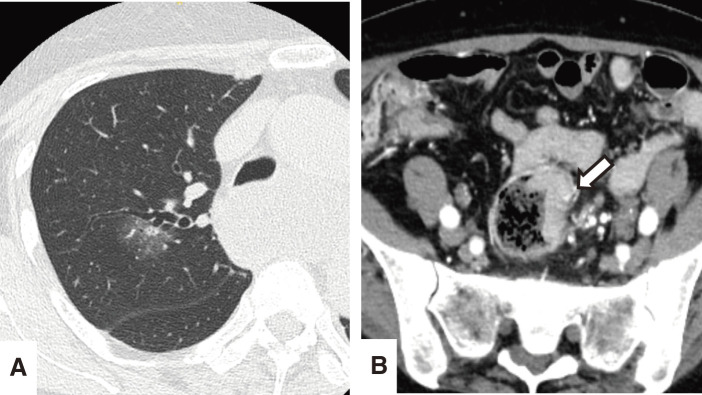
(**A**) A chest CT showing a 26-mm part-solid nodule in the right upper lobe. (**B**) An abdominal CT showing an irregular wall thickening in the sigmoid colon (arrowhead), which was later diagnosed as advanced colon cancer.

Based on these imaging findings, the differential diagnosis included a bronchogenic or parathyroid cyst, and elective surgery was planned. There were safety concerns regarding the risk of tracheobronchial injury caused by a double-lumen tube intubation and circulatory failure due to tracheal compression by the cyst during the induction of general anesthesia.^12,13)^ In addition, a whole-body screening CT incidentally identified operable advanced sigmoid colon cancer and primary lung cancer in the right upper lobe (**Fig. 4**). For oncological reasons, surgery for sigmoid colon cancer was prioritized. After consulting with the anesthesiology team, it was decided to initially perform needle aspiration of the mediastinal cyst to secure the airway, followed by prioritizing the colorectal cancer surgery. A simultaneous right upper lobectomy and mediastinal cystectomy were then planned. To mitigate those risks, we planned to perform an EUS-FNA to reduce the cystic volume prior to surgery.

**Fig. 5:**
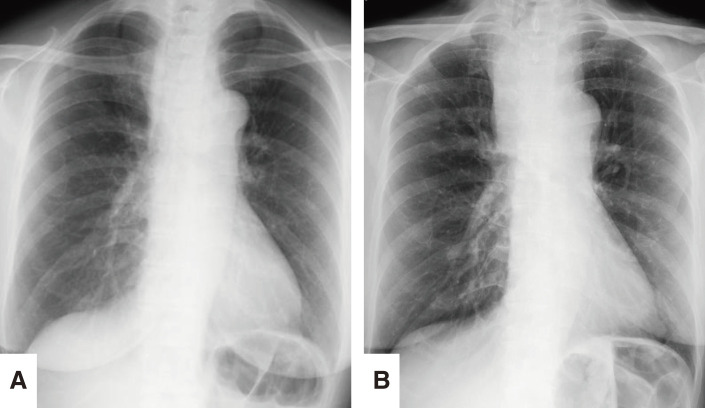
(**A**) A chest radiograph immediately after the EUS-FNA showing a normal mediastinum. (**B**) A chest radiograph 3 days after the EUS-FNA showing recurrent mediastinal widening. EUS-FNA, endoscopic ultrasound-guided fine-needle aspiration

EUS revealed a hypoechoic area measuring 70 mm × 35 mm adjacent to the ventral esophagus. An FNA was performed using an EZSHOT 19 G needle (Olympus, Tokyo, Japan), aspirating a total of 125 mL of serous, yellowish cystic fluid without any bloody components. This resulted in a reduction in cyst size, relief of the tracheal compression, and improvement in the respiratory distress (**Fig. 5A**). The procedure was successfully completed in a single attempt without hemorrhage, and no prophylactic antibiotics were administered.

However, 3 days after the EUS-FNA, the patient developed a cough, dyspnea, and difficulty lying supine. A chest radiograph revealed a widened mediastinum (**Fig. 5B**), and contrast-enhanced CT demonstrated a high-density area within the mass, suggesting an intracystic hemorrhage (**Fig. 2B**). As the airway stenosis had worsened compared to the pre-procedure state, we decided to perform an emergency surgery on the same day.

Given concern about a potential airway obstruction during anesthetic induction, which could result in difficult ventilation and intubation, V-A ECMO was prepared as a backup measure. A percutaneous femoral cannulation was performed using 6 Fr sheaths in the right femoral artery and vein under conscious sedation. Anesthesia was induced with 4 mg of remimazolam. Following laryngoscopic exposure, a 7.0-mm tapered-guard endotracheal single-lumen tube was inserted 24 cm into the trachea using a gum elastic bougie to confirm adequate ventilation. Additionally, a 3.0-mm Phycon TCB bronchial blocker (Fuji Systems, Tokyo, Japan) was placed in the right main bronchus to facilitate 1-lung ventilation.

**Fig. 6:**
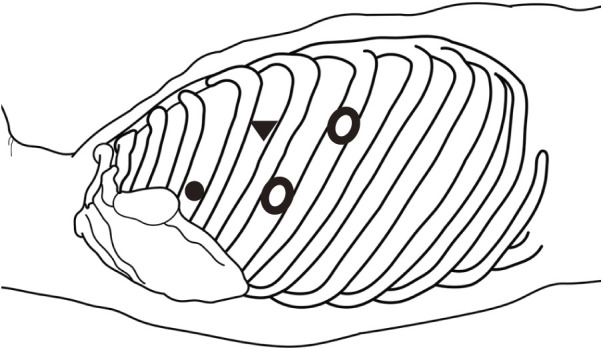
Port insertion sites: 2 cm utility ports for the surgeon’s right hand in the 5th ICS and for the assistant in the 6th ICS (○), 7 mm port for the surgeon’s left hand in the 3rd ICS (●), and an 11 mm camera port in the 4th ICS (▼) were placed. ICS, intercostal space

**Fig. 7:**
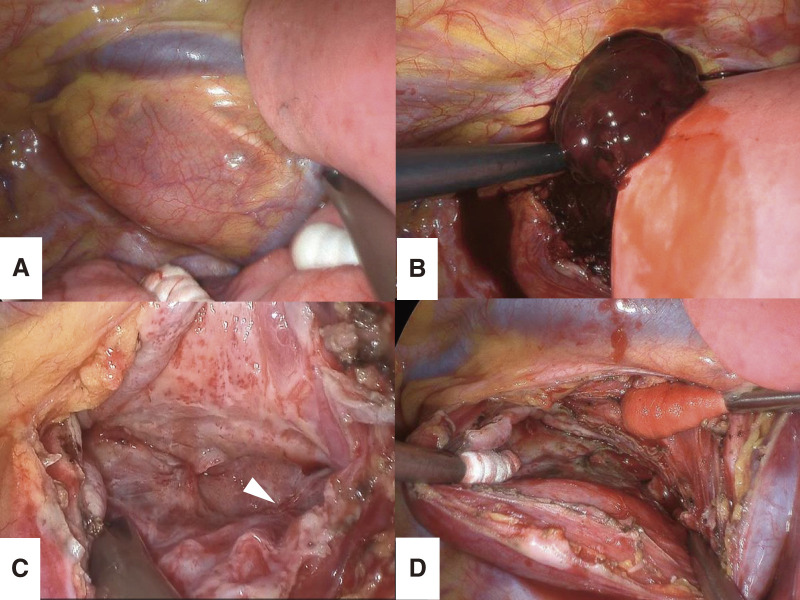
(**A**) The surgical field was established by retracting the upper lobe caudally to expose the expanding cyst in the superior mediastinum, despite the use of 2-lung ventilation. (**B**) The cyst was filled with hematologic fluid and a hematoma. (**C**) The most likely bleeding site was reddened along the cyst wall (arrowhead). (**D**) The cyst wall was maximally incised, and the residual luminal mucosa was cauterized.

With the patient in the left lateral decubitus position, 3 ports were inserted: a 7-mm port in the 3rd intercostal space along the posterior axillary line, a utility port in the 5th intercostal space along the posterior axillary line, and an 11-mm camera port in the 4th intercostal space along the anterior axillary line. An additional utility port was placed in the 6th intercostal space along the anterior axillary line for the assistant, establishing a VATS setup with a facing upside-down monitor configuration (**Fig. 6**). Due to an insufficient lung isolation caused by instability of the blocker occlusion in the right main bronchus, the surgical procedure was performed under 2-lung ventilation with intermittent pauses. The upper lobe was retracted caudally to expose a tense cyst in the superior mediastinum (**Fig. 7A**). The cyst contained hematologic fluid and a hematoma (**Fig. 7B**), with 2 vessels, approximately 1 mm in diameter, running along the cyst wall. While the suspected puncture site was reddened, no active bleeding was observed (**Fig. 7C**). As the cyst wall was tightly adherent to the surrounding organs and the preoperative serum intact parathyroid hormone level was normal, ruling out a functional parathyroid cyst, complete resection of the cyst wall was deemed unnecessary regardless of its origin.^14,15)^ The cyst wall was resected as extensively as possible, and the luminal mucosa of the residual cyst was cauterized (**Fig. 7D**). The operative time was 92 minutes, with a blood loss of 25 g. The postoperative course was uneventful, with no rebleeding, and the patient was discharged on postoperative day 2.

**Fig. 8:**
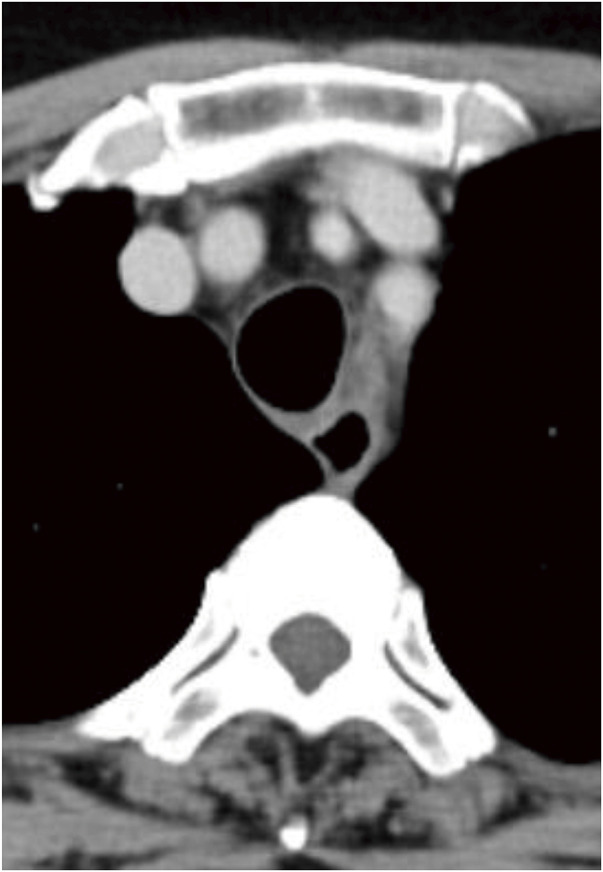
Contrast-enhanced CT at 3 months after surgery showing no evidence of recurrence.

A histopathological analysis revealed fibrous connective tissue with lymphocyte and plasma cell infiltration in the cyst wall. Immunostaining showed only a few CKAE1/AE3-positive cells within the cyst lumen; however, most of the epithelium had detached, preventing the determination of the cyst’s origin. Additionally, PAX8, WT1, and ER were all negative, with no findings suggestive of a Müllerian duct origin. Although a definitive pathological diagnosis could not be established, the cyst’s location and clinical course were consistent with a bronchogenic cyst. No recurrence was observed at the 3-month postoperative follow-up (**Fig. 8**).

## DISCUSSION

This case provided 2 important insights. First, a delayed intracystic hemorrhage can occur after an EUS-FNA of mediastinal cysts. Second, VATS using a confronting upside-down monitor configuration is useful for superior mediastinal tumors, even in cases where a lung isolation is insufficient.

Bronchogenic cysts are rare congenital malformations originating from abnormal foregut development.^4)^ Most cases remain asymptomatic unless an intracystic infection develops or the cyst compresses adjacent mediastinal structures.^1)^ However, although rare, a malignant transformation and hemorrhage have also been reported.^16,17)^ Therefore, a prophylactic surgical resection is recommended, even in asymptomatic cases, to prevent these complications and avoid increased surgical difficulty once symptoms develop.^4,18)^ In cases with severe adhesions or conditions where a complete resection is not mandatory—except for cases requiring the total removal, such as functional parathyroid cysts—a palliative fenestration with cauterization of the residual luminal epithelium is a feasible alternative.^4,14)^

In recent years, less invasive techniques, such as transbronchial or transesophageal aspiration and CT- or ultrasound-guided percutaneous aspiration, have been reported as alternative approaches.^19–22)^ These techniques are associated with a lower complication rate and may serve as both a preoperative volume reduction and, in selected cases, a definitive treatment.

The ESGE technical guidelines report that an EUS-FNA is generally a safe procedure with an approximate 1% complication rate.

The risk of bleeding is low, ranging from 0% to 0.5%, and rarely poses a clinical issue. Although the ESGE guidelines also acknowledge the potential utility of an EUS-FNA in mediastinal cystic lesions, they do not recommend its use for simple cysts due to the risk of mediastinitis.^23)^

Nevertheless, we considered the procedure essential for reducing the cystic volume preoperatively to prevent an airway obstruction and circulatory failure during anesthesia induction. Despite an initial transient reduction in the size, the delayed intracystic hemorrhage ultimately worsened the airway obstruction, necessitating emergency surgery with V-A ECMO on standby. These findings underscored the importance of recognizing the risk of delayed bleeding following an EUS-FNA for mediastinal cysts. While some studies have reported that neither the number of punctures nor the needle gauge significantly influences the risk of complications, others emphasize the safety of a single puncture and suggest that a 22 G or smaller bore needle is sufficient for aspirating simple cysts.^23–25)^ In the present case, even a single puncture with a 19-G needle triggered critical bleeding, suggesting that a finer gauge needle should have been used. If daily observation had been continued beyond the day following the puncture, the current condition might have been detected at an earlier stage.

Tracheal intubation was successfully performed without the need for circulatory support. However, to minimize the risk of tracheobronchial membranous injury associated with a double-lumen tube, a single-lumen tube was selected. That decision resulted in inadequate lung isolation, necessitating surgery under intermittent respiratory arrest.^12)^ Despite those challenges, the VATS procedure was successfully completed, largely due to the implementation of a confronting upside-down monitor setting.^26)^

The VATS using a confronting upside-down monitor configuration has recently been applied not only to lung cancer surgery but also to a mediastinal tumor resection.^27)^ Individual display screens, one of which is inverted for the operator and assistant, resolve the mirror-image issue, facilitating natural manipulation comparable to conventional chest surgery. This approach provides the shortest camera-to-target distance, ensuring a magnified field of view with fewer interposing structures, even in narrow and deep surgical fields, compared to the conventional look-up method. For lesions in the superior mediastinum, as in the present case, this configuration offers superior visibility over the conventional look-up approach.

In this case, the confronting upside-down monitor configuration was especially advantageous due to the lesion's location in the superior mediastinum and the inability to achieve 1-lung ventilation. The camera and the operator’s instruments assessed the lesion via the shortest distance, allowing the procedure to be performed even under 2-lung ventilation with only minimal lung retraction by the assistant to maintain the surgical field. Had the conventional look-up monitor configuration been used, visualization of the superior mediastinum would have required access from a lower intercostal space, significantly compromising the maneuverability over the ventilated lung. Additionally, the close-up visualization allowed the precise identification of the intracystic vessels and suspected puncture sites, facilitating a comprehensive observation without blind spots. In the present case, the camera port positioned in the higher intercostal space played a crucial role in providing an optimal surgical field and enhancing operative maneuverability. These findings suggest that VATS using a confronting upside-down monitor configuration may be a feasible option not only for anatomical lung resection but also for superior mediastinal tumor resection, particularly in cases where intraoperative visualization is challenging.

## CONCLUSIONS

We should be aware of the risk of a delayed intracystic hemorrhage and the associated risk of an airway obstruction after an EUS-FNA for a mediastinal cyst. VATS with a confronting upside-down monitor configuration provides sufficient maneuverability even in cases with a limited surgical field, particularly for superior mediastinal tumors where 1-lung ventilation is challenging. Placement of the camera port in the higher intercostal space was deemed particularly crucial.

## ACKNOWLEDGMENTS

We thank Mr. John Martin for his proofreading of the manuscript.

## DECLARATIONS

### Funding

No funding was received for this study.

### Authors’ contributions

EU wrote this paper.

YO reviewed the pathological findings.

TN reviewed and edited the manuscript.

All authors read and approved the final manuscript.

### Availability of data and materials

Not applicable.

### Ethics approval and consent to participate

This work does not require ethical considerations or approval. Informed consent to participate in this study was obtained from the patient.

### Consent for publication

Written informed consent for the publication of the case details was obtained from our patient.

### Competing interests

The authors declare that they have no competing interests.
